# The correlation between white matter integrity and pragmatic language processing in first episode schizophrenia

**DOI:** 10.1007/s11682-020-00314-6

**Published:** 2020-07-24

**Authors:** Agnieszka Pawełczyk, Emila Łojek, Natalia Żurner, Marta Gawłowska-Sawosz, Piotr Gębski, Tomasz Pawełczyk

**Affiliations:** 1 Department of Affective and Psychotic Disorders, Medical University of Łódź, Łódź, Poland; 2 University of Warsaw, Warsaw, Poland; 3 Adolescent Ward, Central Clinical Hospital of Medical University of Łódź, Łódź, Poland; 4 SYNAPSIS Foundation, Warsaw, Poland; 5 Scanlab Diagnostyka Medyczna Księży Młyn, Medical Examination Centre, Medical University of Łódź, Łódź, Poland

**Keywords:** Diffusion tensor imaging, First episode schizophrenia, White matter, Pragmatic language, Extralinguistic skills

## Abstract

**Objective**: Higher-order language disturbances could be the result of white matter tract abnormalities. The study explores the relationship between white matter and pragmatic skills in first-episode schizophrenia. **Methods:** Thirty-four first-episode patients with schizophrenia and 32 healthy subjects participated in a pragmatic language and Diffusion Tensor Imaging study, where fractional anisotropy of the arcuate fasciculus, corpus callosum and cingulum was correlated with the Polish version of the Right Hemisphere Language Battery. **Results:** The patients showed reduced fractional anisotropy in the right arcuate fasciculus, left anterior cingulum bundle and left forceps minor. Among the first episode patients, reduced understanding of written metaphors correlated with reduced fractional anisotropy of left forceps minor, and greater explanation of written and picture metaphors correlated with reduced fractional anisotropy of the left anterior cingulum. **Conclusions**: The white matter dysfunctions may underlie the pragmatic language impairment in schizophrenia. Our results shed further light on the functional neuroanatomical basis of pragmatic language use by patients with schizophrenia.

## Introduction

Although there is the body of evidence suggesting that language dysfunctions play a role in schizophrenia (Kuperberg 2010a, 2010b; Bryan 2014; Pienkos and Sass 2017; Zimmerer et al. 2017), only a few studies have examined the wide range of higher-order language (pragmatic) processing in this disease (Bosco and Parola 2017; Bambini et al. 2016; Colle et al. 2013; Pawelczyk et al. 2017; Pawelczyk et al. 2018b; Pawelczyk et al. 2018a). These pragmatic aspects of language refer to various process and features of speech: lexical-semantic processes, understanding and production of vocal non-verbal speech components (prosody), the comprehension of indirect speech acts, non-literal language (idioms, metaphors, irony), discourse comprehension and production, as well as the appreciation of shared knowledge and reflection. They allow the context of interaction to be taken into account, and regulate communicative exchange by a developed and shared system of rules (Łojek 2009; Balconi 2010; Bryan 1995; Bambini et al. 2016). Studies suggest that disturbances in pragmatic processing may be related to the cognitive dysfunctions typically displayed by schizophrenia patients (Docherty 2012; Bosco et al. 2012); however, while some suggest that cognitive and executive functions influence language use (Docherty 2005; Gavilan and Garcia-Albea 2011) others do not (Parola et al. 2017). Such disturbances can present serious obstacles in everyday communication. In particular, they can hamper social communication by obscuring the intentions and emotions of others, making it difficult for the listener to keep track of the topic of conversation or to comprehend inferred meanings. In speech production, they lead to a greater focus on minutiae, interjecting inappropriate remarks or omitting important information, thus making it difficult to convey a message or an intention (Joanette et al. 2008; Jodzio et al. 2005; Myers 2001; Tompkins et al. 2008).

Pragmatic language processing requires both linguistic and non-linguistic skills, and therefore is likely to be supported by cortical structures in both hemispheres. Studies on neural correlates suggest that such language processes are mainly associated with the frontal, temporo-parietal and cingular corticies; these processes often act bilaterally, with the right hemisphere appearing to play the dominant role (Beaty et al. 2017; Babajani-Feremi 2017; Ilie et al. 2017; Joyal and Fecteau 2016; Prat et al. 2011; Price et al. 2016; Rapp et al. 2012; Catani and Bambini 2014; Hagoort and Levinson 2014). However, since language processing depends not only on cortical brain regions but also on the white matter bundles that connect the cortices (Zaidel et al. 2003; Saur et al. 2008; Saur et al. 2010; Caplan 2003) and both hemispheres are involved in these complex processes, it could be supposed that the white matter tracts linking fronto-temporal, temporo-parietal cortices (Clark et al. 2012; Kubicki et al. 2013; Catani and Bambini 2014), cingulated cortex (Bambini et al. 2011) and the corpus callosum possibly play important roles in pragmatic language processing in patients with schizophrenia.

Unfortunately, studies on higher-order language functions and their white matter correlates remain very limited. While arcuate fasciuculous involvement has been noted in all aspects of language, it appears to be predominantly associated with language production (Catani and Budisavljevic 2014; Ivanova et al. 2016; Caplan 2003) and phonological processing (Sarubbo et al. 2015). However, some models also suggest a possible relationship between abnormalities in the anatomy of the posterior segment of the arcuate fasciculus (Catani et al. 2011) and impaired pragmatic use of language (Catani and Bambini 2014).

Earlier studies suggest the presence of deficits in paralinguistic pragmatic language functions, in commissurotomy patients: impoverishment of verbal description of emotional experiences, poor comprehension of written paragraphs and conversational interaction, social inappropriateness of behavior, weak understanding of figurative and idiomatic meanings, and impaired story retelling, emotion recognition, nonliteral language and discourse (Zaidel et al. 2003; Spence et al. 1990). These problems have also been noted in children with spina bifida and agenesia and hypoplasia of the corpus callosum (Huber-Okrainec et al. 2005), in males with agenesia of the corpus callosum (Paul et al. 2003), children with agenesia of the corpus callosum (O’Brien 1994; Stickles et al. 2002; Brown and Paul 2000; Brown et al. 2005a; Brown et al. 2005b) and premature births (Reidy et al. 2013). These findings suggest that the corpus callosum may be involved in pragmatic language use.

Earlier animal studies have identified involvement of the cingulum bundle in social responsiveness and affect processing, while studies with cingulotomy patients have suggested that it may also take part in the processing of emotions (Bubb et al. 2018). More recent studies examining the association between emotional prosody and white matter tracts, particularly those based on Diffusion Tensor Imaging, have identified significant relationships between the cingulum bundle and emotional prosody in patients with and without traumatic brain injury (Schmidt et al. 2013). Furthermore, the processing of affective prosody has also been linked with extension of the temporo-frontal network and right ventral auditory pathway (Fruhholz et al. 2015). In addition, a study of metaphor processing identified greater activity of the inferior frontal gyri, right superior temporal gyrus, left angular gyrus and anterior cingulated cortex during metaphors processing (Bambini et al. 2011), and the cingulum bundle was shown to interconnect the frontal, parietal and cingulated cortices (Bubb et al. 2018).

According to the neurodevelopmental hypothesis of schizophrenia, the presence of frank illness in adolescence and early adulthood is associated with impairments in the development of the nervous system. Indeed, such structural brain pathologies have been observed in neuroimaging studies (Owen et al. 2011), and recent studies on white matter tracts have revealed reduced white matter integrity in both first episode patients and subjects at ultra-high risk of psychosis (Karlsgodt et al. 2009; Kubicki et al. 2013; Wheeler and Voineskos 2014). In addition, these findings suggest that alternations in cerebral connectivity may be caused by dysfunctions in white matter integrity. A number of studies suggest that white matter abnormalities may also play a significant role in schizophrenia pathophysiology (Wheeler and Voineskos 2014; Ribolsi et al. 2014; Innocenti et al. 2003; Voineskos et al. 2010). Although their results are not always consistent, they show white matter tract alternations in various bundles, with these being most frequently observed in the cingulum bundle, corpus callosum, uscinate fasciculus, arcuate fasciculus, fornix and internal capsule (Wheeler and Voineskos 2014). Some studies, e.g. (Kitis et al. 2012), also suggest an association between fractional anisotropy and schizophrenia symptoms (Herbsman and Nahas 2010b; Cheung et al. 2011), although some variation is observed in these findings.

However, few studies have examined the influence of white matter abnormalities and language on schizophrenia. However, reduced fractional anisotropy has been noted in the arcuate fasciculus of patients with early-onset schizophrenia, but not in the late-onset schizophrenia (Voineskos et al. 2010; de Weijer et al. 2011; de Weijer et al. 2013; Abdul-Rahman et al. 2012; Kubicki et al. 2013)**.** Increased radial diffusivity in the splenum of the left superior and posterior corona radiata, left superior longitudinal fasciculus, inferior longitudinal fasciculus and inferior longitudinal fasciculus have also been observed in language-impaired childhood-onset schizophrenia patients (Clark et al. 2012); however, these studies have tended to assess language performance by evaluating of semantics and grammar, listening, organizing and speaking. Furthermore, while patients with schizophrenia and auditory verbal hallucinations demonstrated bilateral fractional anisotropy reduction in the arcuate fasciculus, those without verbal hallucinations only demonstrated significant differences in the left arcuate fasciculus (Catani et al. 2011). These reductions were found to be specific to connections between the posterior temporal and anterior regions of the inferior frontal and parietal lobes.

While all schizophrenia patients, with or without auditory verbal hallucinations, present increased diffusivities in the interhemispheric fasciculi, only those with auditory verbal hallucinations have decreased fractional anisotropy in the interhemispheric auditory pathway (Leroux et al. 2017). In addition, loss of integrity in the interhemispheric callosal fibers has been associated with reduced leftward cerebral dominance for language in patients with schizophrenia (Leroux et al. 2015), which could contribute to pragmatic dysfunction.

While some studies have linked the state of white matter with various aspects of language in schizophrenia patients, typically comprehension, speaking, listening, semantics and grammar, little is understood of its influence on pragmatic language processing. Previous brain connectivity literature has examined the association between different language aspects, including pragmatics, and arcuate fasciculus (Catani and Budisavljevic 2014; Ivanova et al. 2016; Catani et al. 2011; Caplan 2003; Catani and Bambini 2014), extralinguistic skills and the corpus callosum (Brown et al. 2005a; Brown et al. 2005b; Zaidel 2003) and emotional prosody with the cingulum bundle (Schmidt et al. 2013).

In addition, a large body of research examines the relationship between abnormalities in the arcuate fasciculus, corpus callosum and the cingulum and the occurrence of schizophrenia, e.g. (Kubicki et al. 2013; de Weijer et al. 2011; de Weijer et al. 2013; Wheeler and Voineskos 2014; Fitzsimmons et al. 2014; Oestreich et al. 2017). These findings suggest that schizophrenia may be associated with abnormalities in the fiber tracts interconnecting anatomically and functionally-distinct neuronal networks of higher-order language skills. Additionally, Hinkley et al. suggest that the corpus callosum may contribute to language lateralization (Hinkley et al. 2016), which is believed to be disturbed in schizophrenia subjects (Sommer et al. 2001; Leroux et al. 2015, 2017).

Some studies indicate that while the right hemisphere itself may not be solely responsible for pragmatic language processing, it nevertheless plays a dominant role (Bryan 1995; Bryan and Hale 2001; Joanette et al. 2008; Joanette and Goulet 1994; Joanette et al. 1990). Others suggest that interhemispheric communication via the corpus callosum may assist the integration of complex language material from the two hemispheres (Huber-Okrainec et al. 2005; Leroux et al. 2015).

Consequently, the aim of the present study was to determine whether dysfunctions of the arcuate fasciculus (long segment), corpus callosum and cingulum bundle, measured by fractional anisotropy in Diffusion Tensor Imaging, correlate with disorders of pragmatic language processing in first episode schizophrenia patients, and whether they are associated with disease-related symptoms. The study examines whether the presence of previously-demonstrated Diffusion Tensor Imaging abnormalities of the arcuate fasciculus, corpus callosum and cingulum bundle in patients with first episode schizophrenia are related to their performance on a higher-order language test battery.

## Methods

### Participants

The participants were divided into two groups: one group experiencing first episode of schizophrenia (*n* = 34) and another group of healthy controls (HC; *n* = 32) (see Table 1). The patients met the ICD – 10 criteria for schizophrenia evaluated by a psychiatrist. The patients were clinically stable, i.e. they had been on a stable regime of antipsychotic therapy for the treatment of schizophrenia with a change in the Clinical Global Impression-Severity (CGI-S) (Guy 1976) score of ≤1 for six or more weeks prior to enrolment. The background antipsychotic therapy and concomitant medications were chosen and titrated according to the Polish standards of pharmacotherapy of mental disorders (Jarema 2015). The daily doses of antipsychotics used were converted into chlorpromazine equivalents using an equivalency table provided by Gardner et al. (Gardner et al. 2010) to enable medication regimes to be compared. The average chlorpromazine equivalent dose for schizophrenia patients is given in Table 1.

**Table 1:** Characteristics of the participants

Variable	HC *n* = 32	FE *n* = 34	Test statistics (df)	*p* Value
Age [years] Mean (SD)	20.21 (4.45)	20.85 (4.27)	-0.5903^a^ (64)	0.5570
Sex, n (%)			0.0002^b^	0.9880
Woman	17 (53.13%)	16 (47.06%)		
Man	15 (46.88%)	18 (52.94%)		
Education (years) Mean	13.03	12.73	0.3765^a^ (64)	0.7077
Right-Handedness, n (%)	32 (100%)	34 (100%)	0.9556^b^ (1)	1.0000
CPZ – chlorpromazine equivalent dose mean (SD)	N/A	263.16 (128.76)		
*PANSS subscales*				
Mean (SD)	N/A			
PANSS p	-	19.44 (7.12)		
PANSS n	-	22.29 (4.8)		
PANSS g	-	43.00 (8.49)		
PANSS	-	84.61 (15.84)		

*FE* First Episode of Schizophrenia, *HC* Healthy Controls, *PANSS* Positive and Negative Syndrome Scale, *PANSS p* positive symptoms subscale of PANSS, *PANSS n* negative symptoms subscale of PANSS, *PANSS g* general psychopathology subscale of PANSS, *df* degrees of freedom, *p Value* two-sided, asymptotic probability of the relevant statistics, *N/A* not applicable; *CPZ* chlorpromazine

^a^t-Student statistics

^b^Chi^2^ – Chi squared test statistics

*significant difference

The inclusion criteria for the healthy control group comprised no psychiatric history, no family history of psychiatric illness, no somatic disease, no traumatic brain injury or any other neurological disease and no history of drug usage. All members of the healthy control group were confirmed as mentally healthy by psychiatric evaluation with the use of the Polish version of the Mini International Neuropsychiatric Interview – M.I.N.I. (Sheehan et al. 1998). This healthy control group was included in this project only to identify impairments in white matter tracts in patients with first episode of schizophrenia when compared to healthy controls, to establish “a norm” and find our regions of interest for this study.

For all participants, the exclusion criteria comprised a history of neurological or chronic somatic disorder, head injury, alcohol or substance abuse or dependence. All individuals were matched according to sex and age. All participants were right-handed, Caucasian, native speakers of Polish, and of Polish ethnicity. Demographic information for all participants and clinical information for patients can be found in Table 1.

### Procedure

#### Language evaluation

On enrollment to the study, all first episode participants were tested during one session with a clinical neuropsychologist. Language and communication processes were assessed by the Right Hemisphere Language Battery by E. Łojek (2007, 2009; Bryan 1995). The battery was chosen for the study because it measures higher-order language functions and has been validated and standardized on a Polish population (Cummings 2009, 2017; Łojek 2007). In addition, it is commonly used to assess pragmatic language in various populations, e.g. those with dyslexia (Cappelli et al. 2018). The Right Hemisphere Language Battery comprises eight tests: Inferential Meaning, Lexical-Semantic, Written Metaphor, Picture-Metaphor, Humor, Emotional Prosody, Linguistic Prosody and Discourse Analysis.

In the Inferential Meaning test, an examinee responds to four questions checking the comprehension of implicit information given in each of four narratives. In the Lexical-Semantic test, a participant is asked to point to the drawing representing the target item named by the examiner; for each item, there are five additional pictures associated with the item: two semantic co-ordinates, a functional associate, phonological and visual controls. In the Humor test, the subject chooses the correct punchline for 10 jokes. Apart from the correct meaning, the responses include a straight ending of neutral content and surprise ending irrelevant to the body of the joke. The number of inappropriate remarks and comments made by the examinee during the Humor and Inferential Meaning tests are noted (the Commentary test). In the Written Metaphor test, the examinee listens to a metaphorical sentence and is asked to chose a correct explanation from three sentences representing possible meanings: a correct metaphorical meaning, a literal meaning and an inappropriate meaning; the subject is then asked to give a personal interpretation of the metaphor, and answers are classified as correct, abstract incorrect or concrete incorrect (the Written Metaphor Explanation test). In the Picture Metaphor test, the participant is asked to point to the picture that matches the meaning of the metaphor read by the examiner. There are four pictures in each set: one representing the correct metaphorical meaning, another the literal meaning, and two control pictures depicting one aspect of the sentence. The accuracy of the explanation of the metaphors by the examinee is also assessed in the Picture Metaphor Explanation test.

In the Polish version of the Right Hemisphere Language Battery, prosody tests comprise 15 nonsense sentences read and recorded by a professional speaker. The sentences are read randomly with three emotional tones (happy, angry and sad) for the Emotional Prosody test, and then are read randomly with intonations expressing statement, question and order for the Linguistic Prosody test. After listening to each sentence, the subject points to the written name of the emotions or linguistic intonations. The Prosody tests are presented from CD. Finally, the Discourse Analysis test evaluates two-way interaction or conversation and appropriateness of behavior in communication settings.

#### Diffusion tensor imaging

The first episode and healthy control groups were evaluated with the Diffusion Tensor Imaging (Emsell et al. 2016), which is a method assessing the microscopic movements of water molecules (Brownian’s motions) in living brain tissues. It is based on the principle that water moves preferentially along axonal bundles: the scalar measure of anisotropy reflects the degree to which diffusion is directionally-dependent (Mori and Barker 1999). Water movement analysis indirectly indicates the degree of density and coherence of brain tissue components (cell membranes, axons, organelles). Diffusion Tensor Imaging data can be used to perform tractography within the white matter to track a fiber along its whole length. Tractography allows deficits in white matter to be identified and measured, and its estimation of fiber orientation and strength is increasingly accurate; the procedure has widespread potential implications in the fields of cognitive neuroscience and neurobiology. Diffusion Tensor Imaging has become a central technique for the study of human brain connectivity, offering easy availability, a relatively short acquisition time and anisotropy measures which can be employed in clinical studies and diagnosis (Maier-Hein et al. 2017). It has also become the most popular technique for investigating white matter abnormalities in neuropsychiatric disorders, including schizophrenia (Kelly et al. 2018; Moseley et al. 2002).

A few measures can be obtained from Diffusion Tensor Imaging analysis: Fractional Anisotropy – a measure of white matter integrity, Trace – a measure of overall diffusion, Axial Diffusity – a measure of diffusion along the neuronal axons and Radial Diffusity – a measure of diffusivity perpendicular to the axons. To analyze Diffusion Tensor Imaging scans, several different methods are used. The region of interest method and tractography reconstructions allow a limited number of regions of interest or specific white matter bundles to be quantified, while voxel-based methods, including voxel-based analysis and more recently tract based spatial statistics (Smith et al. 2006), allow multiple brain regions to be analyzed simultaneously.

All Diffusion Tensor Imaging scans were performed in the NZOZ Diagnostyka Medyczna Księży Młyn (*Księży Młyn Diagnostic Centre*), Łódź, Poland, using a 1.5 T General Electric SIGNA HDi System (GE Medical Systems, Milwaukee, WI) in both groups (first episode and controls). Diffusion-weighed imaging data was acquired with a single-shot echo planar imaging sequence along the anterior–posterior commissure plane. Twenty-seven contiguous axial slices were acquired with a slice thickness of 5 mm and no gap, with diffusion sensitizing gradients applied along 25 nonparallel directions (b = 1000 s/mm2) and the other two acquired without diffusion weighing (b = 0). The acquisition parameters were as follows: echo time (TE) = 103.5 ms; repetition time (TR) = 8500 ms; field of view = 30 cm; number of excitations (NEX) = 1 and matrix = 128 × 128.

Additionally, morphological images were acquired for anatomical determinations. T1 and T2 weighted images were obtained in the sagittal, coronal and axial planes with the following acquisition parameters for the T1 images: TE = 5 ms, TR = 24 ms, NEX = 2, FOV = 26 × 19.5 cm, slice thickness = 1.5 cm and matrix = 256 × 192. T2 sequences were acquired as follows: TR = 3000 ms, TE = 96 ms, NEX = 1, FOV = 26 × 26 and matrix = 256 × 192. The total scan time was less than 30 min. The participant lay still during the assessment and the degree of head movement was minimized with foam padding and a strap across the forehead. All scans were reviewed, and scans with significant artifacts were repeated or discarded.

Diffusion Tensor Imaging data was processed using Functool Diffusion Tensor Imaging software (GE Medical Systems, Milwaukee, WI). After computing the fractional anisotropy images, several regions of interest were defined, which were placed in the white matter tracts using identifiable landmarks on fractional anisotropy images and the Mori magnetic resonance imaging atlas of the human white matter. This stage was performed by a radiologist who was blinded to the neuropsychological diagnosis. The image quality and accuracy of region of interest location was assessed by an experienced radiologist. The correct placement was confirmed by examining the display of the regions of interest on the anisotropy image, as well as on the colored orientation images. To limit the occurrence of eddy-current distortion, images were obtained using only certain sequence parameters. The resulting images were evaluated for the occurrence of artifacts (e.g. Nyquist N / 2 ghost artifact). If artifacts were observed, the sequence was repeated.

#### Psychotic symptoms

In addition, the first episode group was assessed with the Positive and Negative Syndrome Scale (Kay et al. 1987; Rzewuska 2002) to describe the severity of the illness and intensity of the symptoms. The scale constitutes four scales measuring positive and negative syndromes, their differential, and general severity of illness. The Positive and Negative Syndrome Scale was administered by trained psychiatrists at one session with the Right Hemisphere Language Battery.

#### Statistical analysis

Statistical analyses included descriptive and inference methods. The distributions of continuous variables were assessed using the Shapiro-Wilk test. Comparisons of continuous variables between study groups were conducted using the Student’s t test for independent variables. Differences in categorical variables were analyzed using the Chi-square test. The strength of associations between fractional anisotropy results for the target bundles of the white matter with different domains of the Right Hemisphere Language Battery was quantified using Pearson’s correlation coefficient. The clinical significance of the differences was assessed using Cohen’s d (Cohen 1988).

To examine the unique contribution of the bundles that significantly correlated with fractional anisotropy in first episode group, a series of hierarchical regression analyses were performed to compute partial (r^2^p) and semi-partial (r^2^sp) correlations; this allowed significant relationships to be evaluated by partitioning the total variance of the dependent variable (language test score) among the independent variables (Diffusion Tensor Imaging measures, the Positive and Negative Syndrome Scale score) (Cohen and Cohen 1975). The squared partial correlation (r^2^p), representing the proportion of variance of a particular neuropsychological test score shared by a specific Diffusion Tensor Imaging-derived brain region (e.g., right arcuate fasciculus), was removed from both the neuropsychological and Diffusion Tensor Imaging measures, as were the effects of the other Diffusion Tensor Imaging-derived brain regions (e.g., left anterior cingulum bundle) and the Positive and Negative Syndrome Scale; this allowed the proportion of the remaining language variance to be isolated, which was uniquely estimated by the Diffusion Tensor Imaging measure. The square of the semipartial correlation (r^2^sp) estimated the amount of language variance uniquely shared with a particular Diffusion Tensor Imaging measure arcuate fasciculus the effects of other Diffusion Tensor Imaging measures on that particular measure and the Positive and Negative Syndrome Scale score had been removed (Cohen and Cohen 1975); the effects of the other independent variables have been removed from the independent variable but not from the dependent variable.

## Results

### Demographic, clinical and fractional anisotropy of the first episode and control groups

**Fig. 1:**
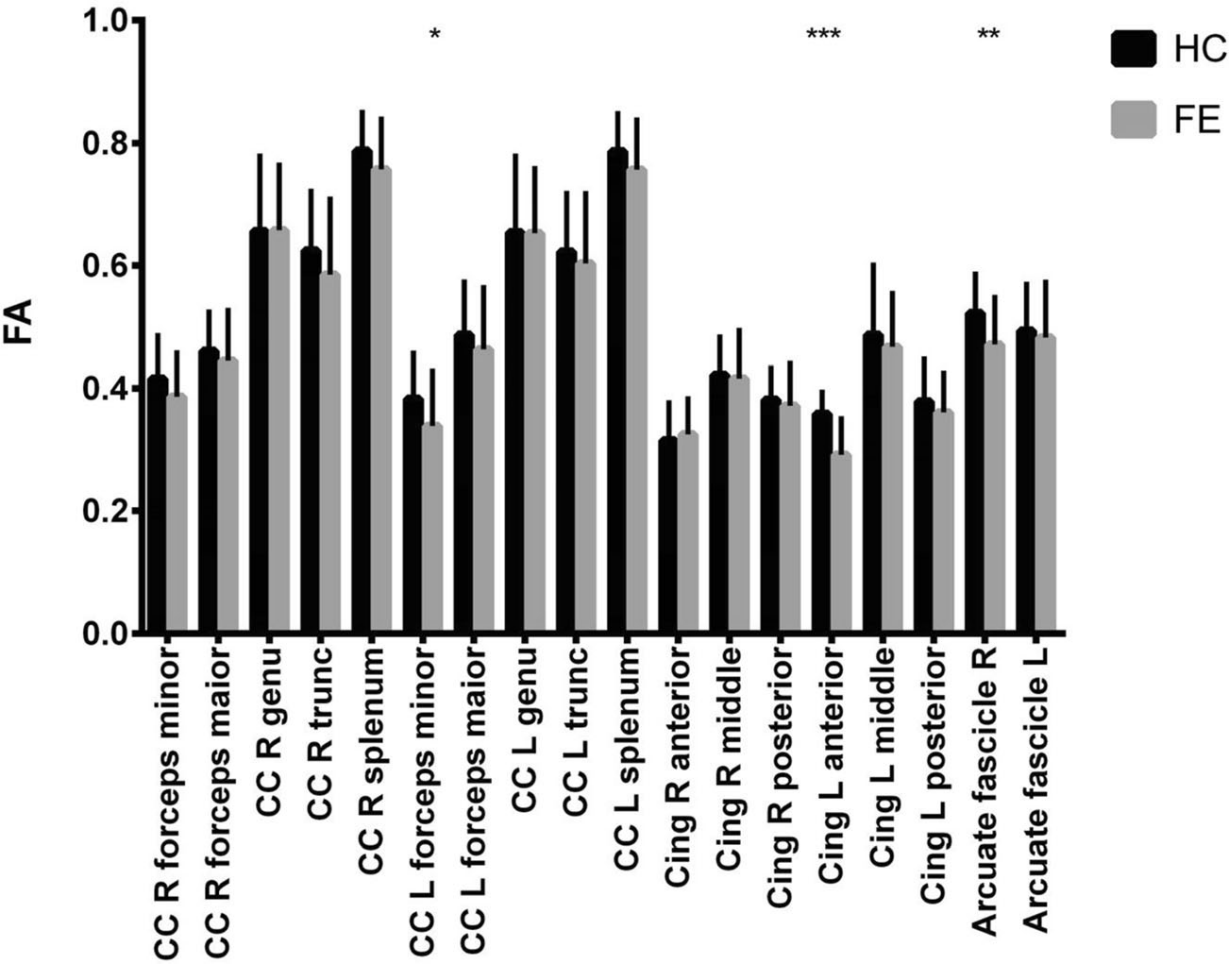
Figure shows fractional anisotropy (FA) comparisons within target white matter bundles of the brain between patients diagnosed with first episode of schizophrenia (FE) and healthy controls (HC). Abbreviations: *FA* fractional anisotropy, *CC* corpus callosum, *R* right, *L* left, *cing* cingulum, *FE* first episode schizophrenia, *HC* healthy controls. Significant differences were marked with asterisks: *p < 0.05, **p < 0.01, ***p < 0.001

**Fig. 2:**
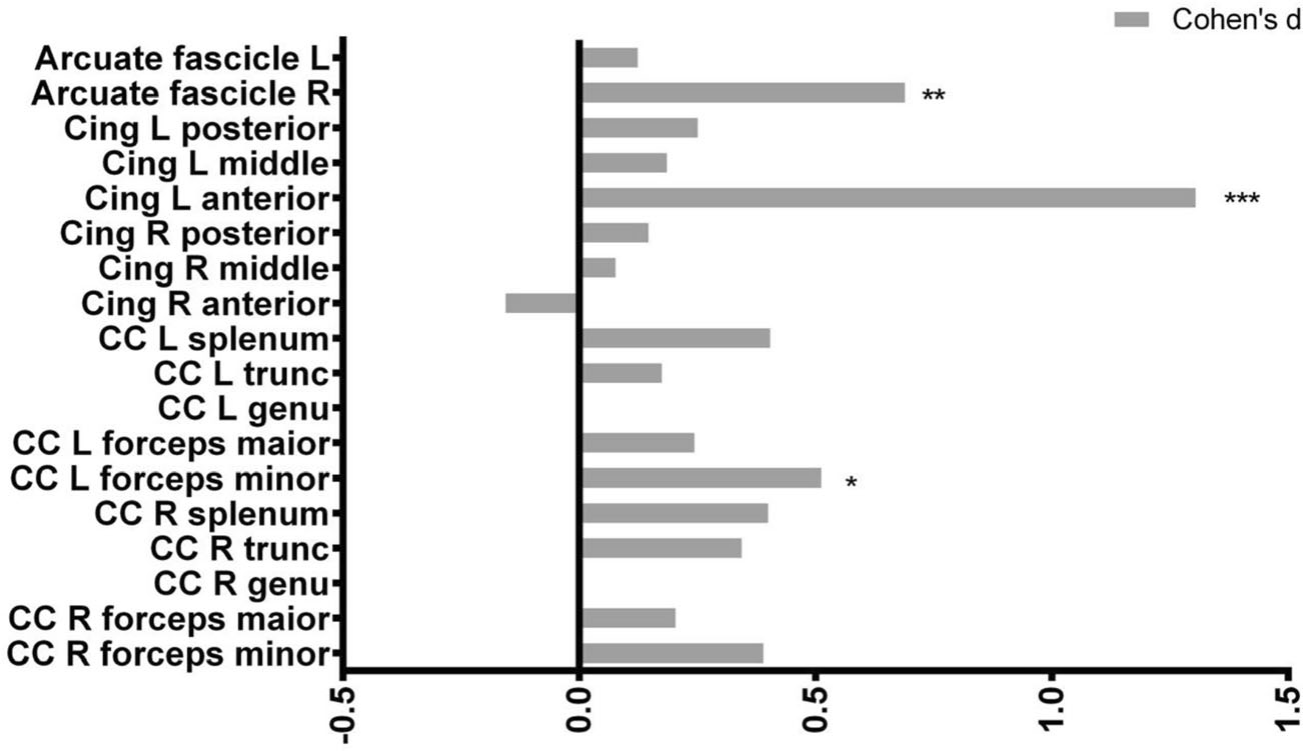
Figure shows Cohen’s d coefficients of the differences in fractional anisotropy (FA) in target white matter bundles of the brain between first episode schizophrenia patients (FE) and healthy controls (HC). Abbreviations: *CC* corpus callosum, *R* right, *L* left, *cing* cingulum, *FE* first episode of schizophrenia, *HC* healthy controls. Significant differences were marked with asterisks: *p < 0.05, **p < 0.01, ***p < 0.001

Demographic and clinical characteristics are summarized in Table 1. The assessed groups did not differ with regard to age, sex or years of education; however, they varied significantly in terms of fractional anisotropy of the right arcuate fasciculus, of the left anterior cingulum bundle of the left forceps minor of the corpus callosum. The results of fractional anisotropy comparison are presented in Fig. 1 and Cohen’s d in Fig. 2.

#### Correlations between decreased diffusion tensor imaging fractional anisotropy and the positive and negative syndrome scale results in first episode schizophrenia patients

In the first episode schizophrenia group, significant correlations were found between fractional anisotropy of the left anterior cingulum (*r* = −0.5034, *p* = 0.002) and left forceps minor (r = −0.4090, *p* = 0.016) and the Positive and Negative Syndrome Scale positive results; correlations were also found between fractional anisotropy of the left anterior cingulum bundle (r = −0.3821, *p* = 0.026) and the Positive and Negative Syndrome Scale total score.

**Fig. 3:**
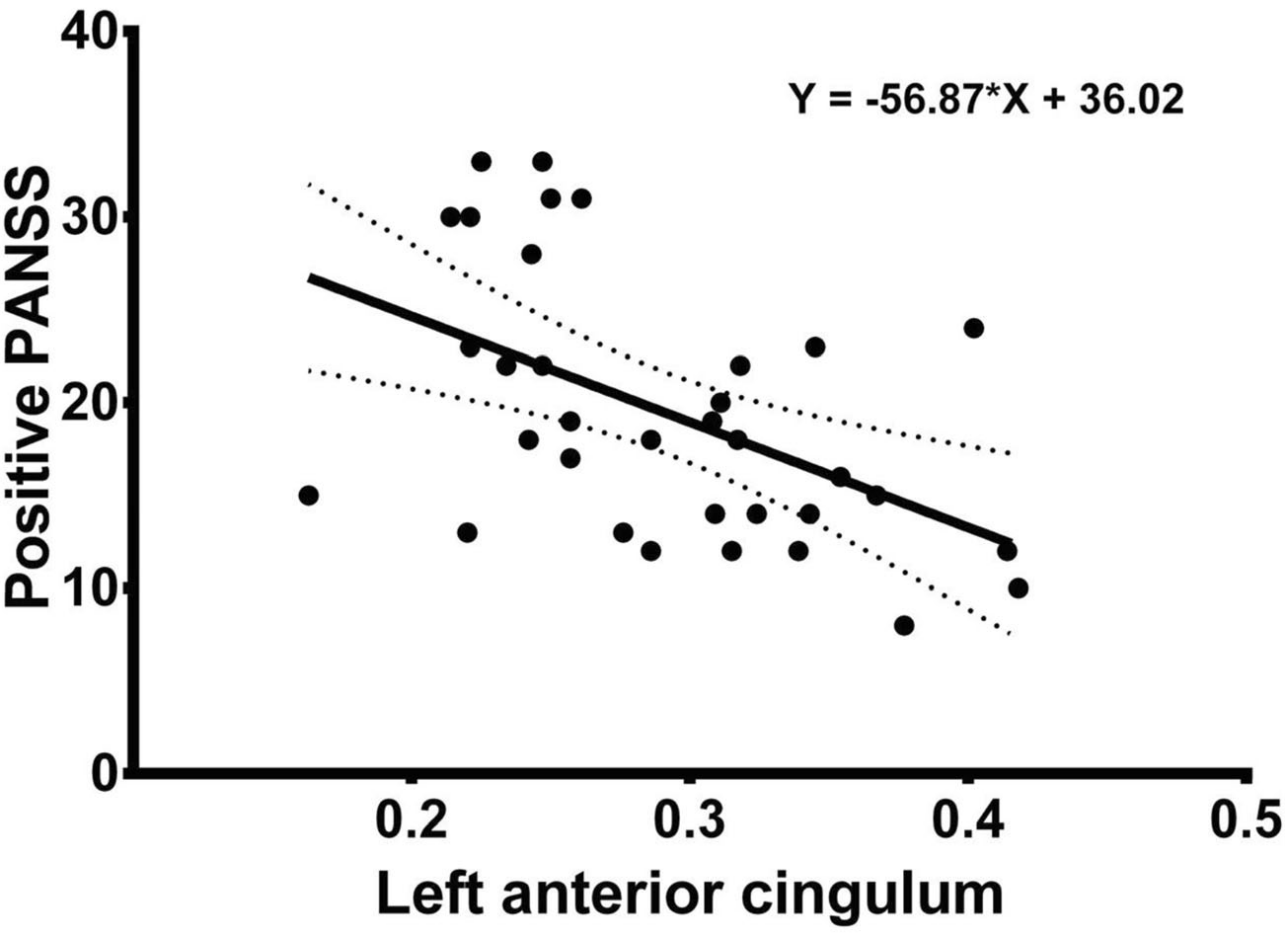
The figure presents the correlation between factional anisotropy of the left anterior cingulum and positive PANSS subscale results

**Fig. 4:**
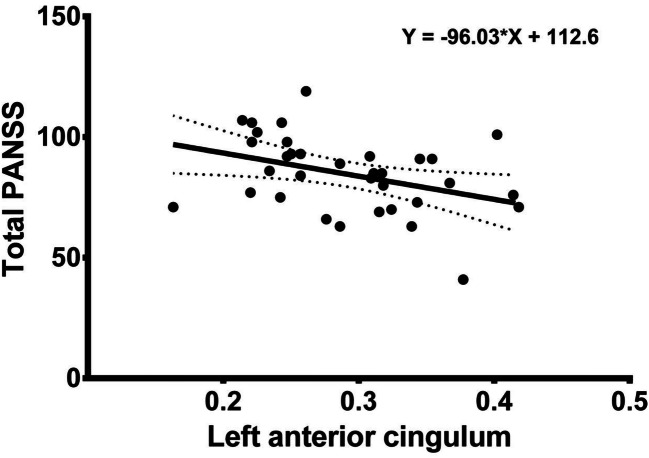
The figure presents the correlation between factional anisotropy of the left anterior cingulum and total PANSS subscale results

**Fig. 5:**
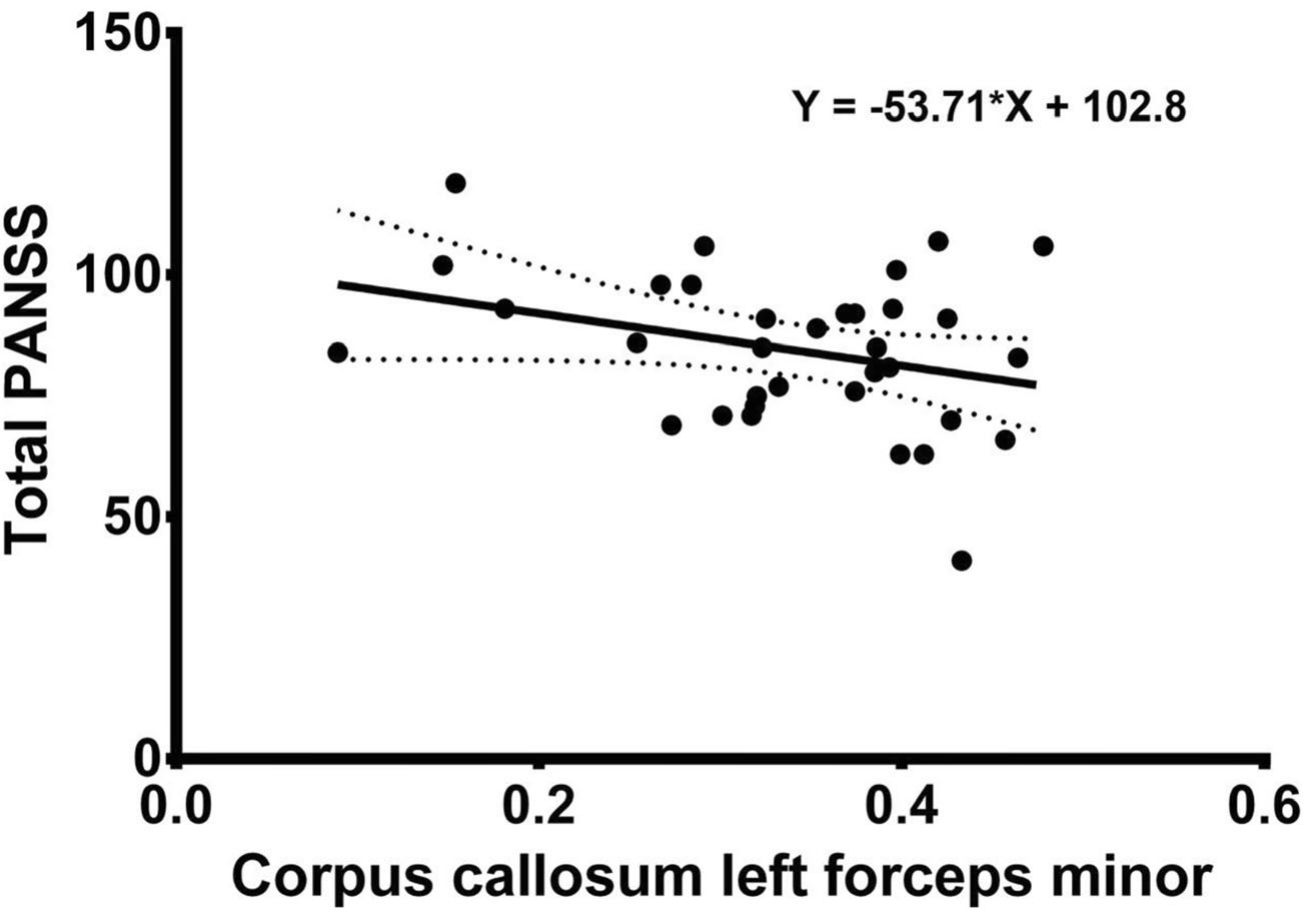
The figure presents the correlation between factional anisotropy of the left forceps minor and total PANSS subscale results

No significant correlation was seen between fractional anisotropy of the right arcuate fasciculus and all the Positive and Negative Syndrome Scale results: positive (r = −0.0529, *p* = 0.766), negative (r = −0.1910, *p* = 0.279), general (r = −0.0825, *p* = 0.643), total (r = −0.1281, *p* = 0.470), between fractional anisotropy of the left anterior cingulum bundle and the Positive and Negative Syndrome Scale negative (r = 0.0297, *p* = 0.868) and general (r = −0.3086, *p* = 0.076) scores, and fractional anisotropy of the left forceps minor and the Positive and Negative Syndrome Scale negative (r = −0.0851, *p* = 0.632) and general scores (r = −0.1988, *p* = 0.260). Significant correlations between fractional anisotropy and disease-related symptoms are presented in Figs. 3, 4 and 5. The significant correlation between the Positive and Negative Syndrome Scale positive score and fractional anisotropy of left anterior cingulum bundle can be regarded as large according to Cohen (Cohen 1988), while the remaining significant relationships, i.e. between the Positive and Negative Syndrome Scale positive results and fractional anisotropy of the left forceps minor and between fractional anisotropy of the left anterior cingulum bundle and the Positive and Negative Syndrome Scale total results, can be regarded as moderate (Cohen 1988).

#### Correlations between diffusion tensor imaging fractional anisotropy and right hemisphere language battery in the first episode group

The results of the Right Hemisphere Language Batter in the first episode group are presented in Table 2. Significant positive correlations were found between the results of the Written Metaphors subtest and the fractional anisotropy of right arcuate fasciculus (*r* = 0.4014, *p* = 0.019) and the fractional anisotropy of the left forceps minor (*r* = 0.4908, *p* = 0.003); these findings indicate that a higher result in the written metaphor understanding test is associated with a higher fractional anisotropy score (“improvement of written metaphors understanding correlates with diffusion restricting to one direction – physiological state of white matter”). Fractional anisotropy of the anterior part of the left cingulum bundle negatively correlated with the Picture Metaphor Explanation scores (*r* = −0.4435, *p* = 0.009) and the Written-Metaphor Explanation scores (*r* = −0.3576, *p* = 0.038), indicating that a higher metaphor explanation test result correlates with a lower fractional anisotropy value (“improvement of metaphors explanation correlates with diffusion being unrestricted in all directions – suggest dysfunctions of white matter”). The strength of these significant correlations can be regarded as moderate to large according to (Cohen 1988). No significant correlations were found between fractional anisotropy of the right arcuate fasciculus, the left anterior cingulum bundle or the left forceps minor of the corpus callosum and the following subtests: Inferential Meaning, Lexical-Semanics, Humor, Commentary, Picture Metaphors, Emotional and Linguistic Prosody, Discourse Analysis or Global Result.

**Table 2:** The scores awarded to the RHLB-PL subscales in the study group of first episode schizophrenia patients

RHLB-PL subscales	Group Mean (min-max)	
	FE *n* = 34	Range of the scores in the RHLB-PL subscale
Inferential meaning	13.11 (7–16)	0–16
Lexical-semantic	12.47 (11–13)	0–13
Humor	8.79 (1–10)	0–10
Commentary	0.56 (0–14)	0–14
Picture-metaphor	8.97 (4–10)	0–10
Written metaphor	9.79 (7–10)	0–10
Picture metaphor explanation:	7.97 (5–10)	0–10
Written-metaphor explanation	7.79 (5–10)	0–10
Emotional prosody	12.82 (7–16)	0–16
Linguistic prosody	14.11 (8–16)	0–16
Discourse analysis:	53.97 (30–96)	0–60
Language factor	25.12 (10–30)	0–30
Cognitive factor	18.14 (11–30)	0–30
Perceptual – logical factor	16.91 (11–20)	0–20
Socio – emotional factor	14.44 (7–20)	0–20
Self-control/-restrain factor	9.06 (1–14)	0–10

*FE* Schizophrenia first episode group, *RHLB-PL* Right Hemisphere Language Battery – Polish Version, *min* minimum, *max* maximum

#### Multiple regression analysis of diffusion tensor imaging and the right hemisphere language battery subtest and the positive and negative syndrome scale in the first episode group

It was next tested whether the obtained important correlations may indicate significant relationships between relevant language tests and the presence of fractional anisotropy in the left arcuate fasciculus, left forceps minor or left anterior cingulum bundle; the results were controlled for schizophrenia symptoms measured by the Positive and Negative Syndrome Scale, or for a second brain region (white matter bundle) in one case. A standardized estimate of effect-size was provided (Cohen’s d), and small (d = 0.2–0.5), medium (d = 0.5–0.8) and large effect-size (d > 0.8) metrics were used (Cohen 1988).

First, two brain regions, i.e. the left arcuate fasciculus and left forceps minor, and the Positive and Negative Syndrome Scale positive score were entered as predictors in a hierarchical regression analysis with the Written Metaphor test. The analysis yielded R^2^ = 0.362, meaning that 36.2% of the outcome of the Written Metaphor test measure can be predicted by our model. For the Written Metaphor test, the left forceps minor produced a significant change of R^2^ = 0.096, F(1, 31) = 4.59, *p* = 0.04, the Positive and Negative Syndrome Scale positive score produced a significant change of R^2^ = 0.261, F(1, 32) = 11.28, *p* = 0.01, while the left arcuate fasciculus accounted for a nonsignificant R^2^ change of 0.006, F(1,30) = 0.269, *p* = 0.608. A partial correlation value of 0.36 and semipartial correlation value of 0.31 (d = 0.77) was found between the left forceps minor (M = 0.339, SD = 0.09) and Written Metaphor test (M = 9.8, SD = 0.64); values of −0.39 and − 0.34 for the Positive and Negative Syndrome Scale positive results (M, SD see Table 1) and Written Metaphor test. These values indicated that the left forceps minor accounted for 13% (9.5% uniquely) of the variance in Written Metaphor test scores and the Positive and Negative Syndrome Scale positive score accounted for 15% (11.4% uniquely) of the variance in Written Metaphor test scores; 20.9% of the Written Metaphor test variance was uniquely explained by these two predictors acting individually (Tolerance = 0.833).

Next, the left anterior cingulum bundle, the Positive and Negative Syndrome Scale positive and total scores were entered as predictors in a hierarchical analysis with the Written Metaphor Explanation test. The analysis R^2^ was found to be 0.152, meaning 15.2% of the Written Metaphor Explanation test outcome can be predicted by the model. For the Written Metaphor Explanation test, the left anterior cingulum bundle produced significant change of R^2^ = 0.116, F(1, 30) = 4.09, *p* = 0.05 in contrast to nonsignificant R^2^ change of 0.024, F(1,30) = 0.782, *p* = 0.383 accounted for by the Positive and Negative Syndrome Scale total and positive scores. A negative partial correlation value of −0.35 and semipartial correlation value of −0.34 (d = 0.75) was found for the Left anterior cingulum bundle (M = 0.293, SD = 0.063) and Written Metaphor Explanation test (M = 7.79, SD = 1.67), while 0.06 and 0.05 were found for the Positive and Negative Syndrome Scale positive result and the written metaphor explanation test, and −0.14 and −0.13 for the Positive and Negative Syndrome Scale total score (m, sd see Table 1) and the written metaphor explanation test. These values indicate that the left anterior cingulum bundle accounted for 12% (12% uniquely) of the variance in written metaphor explanation test scores (tolerance = 0.747).

Finally, left anterior cingulum bundle and the Positive and Negative Syndrome Scale positive and total results were entered as predictors in a hierarchical analysis with Picture Metaphor Explanation test. The analysis R^2^ was found to be 0.254, meaning 25.4% of the Picture Metaphor Explanation test outcome can be predicted by the model. For the Picture Metaphor Explanation test, the left anterior cingulum bundle produced an insignificant change of R^2^ = 0.068, F(1, 30) = 2.72, *p* = 0.11as well as the Positive and Negative Syndrome Scale total score R^2^ = 0.004, F(1, 31) = 0.14, *p* = 0.71, in contrast to the significant R^2^ change of 0.183, F(1,32) = 7.16, *p* = 0.012 accounted for by the Positive and Negative Syndrome Scale positive score. Left anterior cingulum bundle (M = 0.293, SD = 0.063) and Picture Metaphor Explanation (M = 7.79, SD = 1.67) revealed an insignificant partial correlation value of −0.29 and a semipartial correlation value of −0.26, as well as insignificant values of 0.19 and 0.18 for the Positive and Negative Syndrome Scale positive score and the Written Metaphor Explanation test and − 0.05 and − 0.04 for the Positive and Negative Syndrome Scale total score and Written Metaphor Explanation test (Tolerance = 0.742).

## Discussion

The aim of the study is to identify any relationship between pragmatic skills in first episode schizophrenia subjects and the integrity of the white matter in the arcuate fasciculus, corpus callosum and cingulum bundle. Although the first episode subjects did not differ from healthy controls with regard to age or education, the first episode patients demonstrated lower fractional anisotropy values for the right arcuate fasciculus, anterior left cingulum bundle and left forceps minor. A review of Diffusion Tensor Imaging studies carried out in schizophrenia patients reveals inconsistent fractional anisotropy results for first episode subjects (Wheeler and Voineskos 2014), with some studies showing differences in the integrity of the white matter tracts of corpus callosum, cingulum bundle and arcuate fasciculus (Lee et al. 2013; Henze et al. 2012; Dekker et al. 2010), while others exhibit no differences (Peters et al. 2008; Luck et al. 2011).

Our findings suggest that the structural integrity of the left anterior cingulum bundle and the left forceps minor of the corpus callosum negatively correlates with schizophrenia-positive symptoms (the Positive and Negative Syndrome Scale positive score), and structural integrity of left anterior cingulum bundle correlates with whole psychopathology (total results of the Positive and Negative Syndrome Scale). No correlation was identified between the characteristics of other bundles and the presence of schizophrenia symptoms measured by positive and negative syndrome scale. Findings concerning the relationship between fractional anisotropy data and schizophrenia symptoms are also inconsistent: a number of studies show a connection between fractional anisotropy and schizophrenia psychopathology (Rotarska-Jagiela et al. 2009; Seitz et al. 2016; Serpa et al. 2017; Ohtani et al. 2015) while others do not (Voineskos et al. 2010; Herbsman and Nahas 2010a; Joo et al. 2017; Psomiades et al. 2016; Wang et al. 2013; Lee et al. 2012).

The current study explores the correlation between the structure of white matter and pragmatic language performance in first episode subjects. It was found that the presence of minor abnormalities in the right and left forceps minor influenced the understanding of written metaphors (Written Metaphor test), whereas the presence of abnormalities in the left anterior cingulum bundle influenced the ability to explain written and picture metaphors (Written and Picture Metaphor tests). In addition, hierarchical regression analysis indicated that the left forceps minor accounted for 13% of the variance in Written Metaphor test scores, and the Positive and Negative Syndrome Scale positive score accounted for 15%; in addition, both predictors acting individually explained 20.9% of the Written Metaphor test variance. Furthermore, hierarchical regression analysis showed that the left anterior cingulum bundle accounted for 12% of the Written Metaphor Explanation test variance uniquely. These results might suggest that abnormalities of the left forceps minor are associated with an understanding of written metaphors and left anterior cingulum bundle disturbances with the ability to explain written metaphors; in cases of FE of schizophrenia, positive symptoms of schizophrenia appear to be associated with comprehension of written metaphors.

Previous studies carried out on commissurotomy patients (Zaidel 2003; Spence 1990) or on children with agenesia and hypoplasia of the corpus callosum (Brown et al. 2005a, b) identified an association between corpus callosum dysfunctions and the understanding of figurative and idiomatic meanings. Some authors hypothesize that the corpus callosum could influence the interpretation of figurative and idiomatic meanings, as well as with resolving conflicts between two alternative meanings (Huber-Okrainec et al. 2005). Impairments in nonliteral language, such as idioms or proverbs, have also been identified in study on males of average intelligence with agenesia of the corpus callosum. The participants demonstrated deficits in self-generated interpretations, but did not exhibit difficulties on the multiple choice items. These findings were interpreted to mean that more complicated tasks require more interhemispheric communication (Paul et al. 2003). In addition, children with agenesia of the corpus callosum were reported by their parent to interpret speech very literally and misinterpret nonliteral language (O’Brien 1994; Stickles et al. 2002).

The results of our study might be regarded as coherent with earlier studies described above indicating an association between corpus callosum and nonliteral language; however, the methods used in the previous studies are very difficult to compare, and their study participants are diagnosed with disorders other than schizophrenia, which makes comparison difficult. As far as neuroimaging is concerned, there are no Diffusion Tensor Imaging studies on nonliteral language to serve as comparison; our results may solely be compared with those of studies on cortical structures, such as those using Magnetic Resonance Imaging, which link metaphor processing to both right and left hemispheres (Papagno et al. 2006; Tompkins 1990; Tompkins et al. 1992; Rapp et al. 2012; Bohrn et al. 2012) especially the frontal (Shibata et al. 2007), temporal (Eviatar and Just 2006) and temporo-parietal cortices (Catani and Bambini 2014). However, as the fibers of the forceps minor allow communication between the frontal lobes, our findings may apply to research on the cortical correlates of metaphor processing. This part of corpus callosum may be a part of neural network linked to metaphors’ processing. As far as the direction of correlation (fractional anisotropy of forceps minor and metaphors’ understanding) is concerned, it is as expected, indicating a relationship between lower fractional anisotropy and a poorer ability to understand metaphors.

The fractional anisotropy of the anterior part of the left cingulum bundle correlated negatively with a capacity to explain written metaphors. The anterior cingulum bundle connects the anterior cingulated cortex and the Prefrontal Cortex in both hemispheres, and probably takes part in attention and executive processes (Nestor et al. 2004; Benes 1993; Orellana and Slachevsky 2013; Michie et al. 2000; Bubb et al. 2018). Unfortunately, no comparable Diffusion Tensor Imaging study has yet been performed, and so it could only be hypothesized that this direction of correlation may be attributed to confounding cognitive dysfunctions (e.g. executive functions, attention) (Lezak et al. 2004) not controlled in this study. On the other hand, one study of metaphor processing showed greater activity in various areas, including the anterior cingulated cortex (Bambini et al. 2011), which is interconnected with other cortices and subcortical nuclei by the cingulum bundle, which might suggest it plays a possible role in processing metaphors (Bubb et al. 2018). Nevertheless, further studies are clearly needed to elucidate the correlation and its direction.

No significant correlations were found between decreased fractional anisotropy of corpus callosum, cingulum bundle and arcuate fasciculus and the Inferential Meaning, Lexical-Semantic, Humor, Commentary, Prosody, Discourse Analysis or Global Result subtest scores. These results may be regarded as inconsistent with those of studies on emotional prosody (Fruhholz et al. 2015; Schmidt et al. 2013; Ilie et al. 2017) which showed a correlation between the integrity of the cingulum bundle and the genu of the corpus callosum and emotional prosody. However, these associations between white matter and emotional prosody have been evaluated in traumatic brain injury patients (Schmidt et al. 2013) or in healthy young adults (Fruhholz et al. 2015) not in first episode of schizophrenia. Based on studies of cortical associations which reveal the involvement of different cortical regions of both hemispheres in humor understanding (Derouesne 2016; Vrticka et al. 2013; Iidaka 2016; Babajani-Feremi 2017), lexical-semantic ability (Code 1997; Grindrod and Baum 2005), inferencing (Prat et al. 2011; Parsons and Osherson 2001; Chow et al. 2008) prosody (Liebenthal et al. 2016; Ilie et al. 2017; Kotz et al. 2003) or discourse (Martin-Loeches et al. 2008; Mason et al. 2008; Kandylaki et al. 2016), it could be assumed that these pragmatic functions would be mediated by and associated with fibers interconnecting the left and right hemisphere; however, no such correlations were obtained in the present study based on first episode of schizophrenia patients. While it is possible that no such correlation exists, it is also possible that the results could have been influenced by the small number of participants or the presence of unevaluated and uncontrolled confounding factors, such as executive functions, attention, level of intelligence, medication or other language skills. Nevertheless, it was not possible to find any studies linking pragmatic language ability to white matter integrity in schizophrenia and specially first episode participants which would allowed comparisons of our data.

Despite years of research, knowledge on the anatomy of human white matter and the relationship between white matter tracts and cognition is still limited. Nethertheless, studies have noted relationships between Diffusion Tensor Imaging abnormalities and neuropsychological results, associating reduced structural integrity with cognitive or language performance (Nestor et al. 2004; Catani et al. 2011; Catani and Bambini 2014). To account for the obtained correlations, the studies suggest that together with the relevant cortical regions, the white matter tracts may form a larger neural system, or network, of cognitive functions, in which disturbances of cortical volume or white matter integrity may influence cognitive or language functions (Moseley et al. 2002; Nestor et al. 2004). The results of our study suggest that fractional anisotropy of the left anterior cingulum bundle and left forceps minor might be associated with metaphor processing. However, as only a small number of participants took part in the study, results generated from hierarchical regression analysis will need to be replicated in larger groups before any firm conclusions can be drawn. It is plausible that these bundles contribute to a diverse range of cognitive functioning and among schizophrenia patients, the effects of reduced fractional anisotropy of the left anterior cingulum bundle and left forceps minor may extend beyond the difficulties experienced on pragmatic language tests. The associations obtained in this research underscore the widely-distributed nature of higher cognition in the brain and the need to avoid formulating simple relationships between function and anatomy. The Diffusion Tensor Imaging results of the current study represent measures of white matter integrity and as such, they cannot account for the underlying mechanisms, i.e. axonal or myelination related changes, and neither can they be equated with the functional connectivity of these pathways. However, the correlations show that metaphor processing may be closely related to the structural integrity of the left forceps minor, linking the frontal cortices of both hemispheres, and the anterior cingulum bundle, linking the anterior cingulated cortex to the prefrontal cortex, and that these cortical regions in turn are thought to be linked to metaphor processing (Bambini et al. 2011). It could be proposed that the cingulum bundle and forceps minor, together with relevant cortical regions, create a network or neural system of metaphor processing; however, this would need further research.

Nevertheless, it is important to emphasize that the correlates identified between neuroanatomical elements and pragmatic skills can only be considered as preliminary findings. They are based on a small sample size of first episode patients, and hence their limited generalisability represents an important consideration which limits the functional – anatomical relationship reported in this study. Although the group is homogenous as far as diagnosis is concerned, it might display some degree of homogeneity with regard to the evolution of the illness, which could influence the study results and limit generalizability to a degree. Also, the number of correlations computed between right hemisphere language battery subtests and brain structure measures increases the risk of committing a Type 1 error of finding a significant correlation by chance: an important limitation of this study. However, in this regard, Cohen notes a few correlations with a moderate level of clinical significance between the white matter content of the left forceps minor and left anterior cingulum bundle and the ability to understand and explain metaphors (Cohen 1988), suggesting that these pathways contribute to the performance of pragmatic language skills. However, in future studies, a correction for multiple correlations should be applied.

Nevertheless, a key strength of this study is that it indicates a possible link between pragmatic skills and white matter integrity in first episode patients, and is one of a very limited number of studies examining pragmatic language performance and white matter; it could therefore be regarded as exploratory/preliminary. Confounding factors such as attention, executive functions, intelligence, language skills and medication were not controlled and hence may have led to the generation of unexpected correlations or even a lack of correlations. Although inconsistent, previous studies suggest that executive and cognitive functions and theory of mind influence pragmatic skills, so it would be worth taking them into account in future studies (Docherty 2012; Parola et al. 2017). Also, medication should be controlled, as it could influence changes in the white matter (Szeszko et al. 2014) as well as language performance (Husa et al. 2017). However, applying a moderate dosage of antipsychotic medication in our study, expressed in terms of chlorpromazine equivalents (Table 1), and the existence of a stable psychiatric state reduces the possible influence of antipsychotic medications on language performance and white matter changes. Additionally, some subtests of the right hemisphere language battery might have been too easy for the participants, leading to lack of correlation because of the sealing effect. Further research with Diffusion Tensor Imaging on larger groups of first episode subjects and white matter tracts which control for confounding factors should be performed. Moreover, research with participation of chronic schizophrenia patients, as well as with people at risk of developing the illness, could clarify the relationship between pragmatic language impairments in schizophrenia and white matter integrity. It could give a greater understanding of language pathology in schizophrenia and may give new insights into brain and white matter pathology in this disease.

## Conclusion

To summarize, a correlation was found between reduced white matter integrity of left forceps minor and left anterior cingulum bundle and a reduced ability to understand and explain written metaphors in patients with first episode of schizophrenia. Although limited and very preliminary, these findings suggest that pragmatic language impairment in schizophrenia might also encompass underlying Diffusion Tensor Imaging measures of the integrity of neural pathways corresponding to specific pragmatic skills. Our results may contribute to the wealth of functional neuroanatomical knowledge concerning the neuropragmatics of schizophrenia by suggesting that white matter tracts may take part in the processing of metaphors. This study opens a new area of research into the correlation of pragmatic language skills with white matter integrity in first episode schizophrenia subjects and thus foster a greater understanding of the functional neuroanatomical basis for processing pragmatic language. It can also shed further light on the disturbances in integration and synchronization of brain networks and neural systems believed to contribute to processing of language. Nevertheless, further studies on the association between pragmatic aspects of language and white matter integrity on different stages of schizophrenia and larger groups are needed to better understand this issue.
